# New Strategies to Optimize Hemodynamics for Sepsis-Associated Encephalopathy

**DOI:** 10.3390/jpm12121967

**Published:** 2022-11-28

**Authors:** Lina Zhao, Bin Liu, Yunying Wang, Zhiwei Wang, Keliang Xie, Yun Li

**Affiliations:** 1 Department of Critical Care Medicine, Tianjin Medical University General Hospital, Tianjin 300052, China; 2 Department of Emergency, Chongqing University Central Hospital, Chongqing Emergency Medical Center, No.1 Jiankang Road, Yuzhong District, Chongqing 400014, China; 3 Department of Critical Care Medicine, Chifeng Municipal Hospital, Chifeng Clinical Medical College of Inner Mongolia Medical University, Chifeng 024000, China; 4 Department of Anesthesiology, Tianjin Institute of Anesthesiology, Tianjin Medical University General Hospital, Tianjin 300052, China

**Keywords:** sepsis-associated encephalopathy, mean arterial pressure, lactate, hemodynamics

## Abstract

**Background**: Sepsis-associated encephalopathy (SAE) is associated with high morbidity and mortality. Hemodynamic dysfunction plays a significant role in the incidence and mortality of SAE. Therefore, this study aimed to explore the relationship between hemodynamic indicators and SAE. **Methods**: 9033 patients with sepsis 3.0 were selected in a prospective study cohort. The LASSO regression model was used to select characteristic variables and remove the collinearity between them. In addition, a generalized additive model was used to find the optimal hemodynamic index value for patients with SAE. Multivariate logistic regression models, propensity matching scores, inverse probability weighting, and doubly robust estimation confirmed the reliability of the study results (i.e., the optimal hemodynamic indicators targeting patients with SAE). **Results**: A mean arterial pressure ≥ 65 mmHg, systolic blood pressure ≥ 90 mmHg, and lactate levels ≤ 3.5 mmol/L decrease the incidence of SAE, whereas a mean arterial pressure ≥ 59 mmHg and lactate levels ≤ 4.5 mmol/L decrease the 28-day mortality in patients with SAE. **Conclusions**: The hemodynamic indices of patients with SAE should be maintained at certain levels to reduce the incidence and mortality in patients with SAE, such that the mean arterial pressure is ≥65 mmHg, lactate levels are ≤3.5 mmol/L, and systolic blood pressure is ≥90 mmHg. These hemodynamic indicators should be targeted in patients with SAE.

## 1. Introduction

Sepsis-associated encephalopathy (SAE) is a diffuse cerebral dysfunction caused by infection [1]. Approximately 70% of the patients with sepsis have brain dysfunction and high mortality rates of about 60% [2,3,4]. The follow-up of patients with SAE after discharge identified a considerable proportion of them had cognitive impairments, such as inattention and memory loss, which seriously reduced their quality of life [5].

Sepsis causes tissue hypoperfusion and metabolic dysfunction, leading to multiple organ disorders [6]. Cerebral perfusion changes are an essential mechanism of SAE. Charalampos Pierrakos et al., found that 50% of the sepsis survivors showed a cognitive decline with a low cerebral blood flow index at discharge [7]. In addition, Christoph S Burkhart et al., and Lucia Rivera-Lara summarized the important role of cerebral perfusion in septic brain dysfunction [5,8]. Normal brain functions depend on an adequate blood supply. However, the mean arterial pressure is low in severe sepsis and septic shock, leading to decreased cerebral perfusion pressure. Additionally, SAE may be secondary to microcirculation dysfunction in the brain with insufficient blood supply. For instance, Young G.B. et al., found that some patients with sepsis may experience brain dysfunction even if the hemodynamics of macrocirculation are adequate [9], which may be attributable to microcirculation dysfunction. Fabio Silvio Taccone et al., found that cerebral microcirculation significantly reduced the perfusion of small blood vessels in sepsis of sheep-brain microcirculation [10]. These findings indicate that both the macrocirculation and microcirculation of hemodynamics play an important role in the brain dysfunction of patients with sepsis.

According to the third international consensus definition for sepsis and septic shock, a mean arterial pressure > 65 mmHg is essential to ensure multiple organ perfusion in patients with sepsis [11]. However, Kathryn Rosenblatt et al., reported a non-linear relationship between mean arterial pressure levels and patients with SAE and that the optimal mean arterial pressure to ensure an appropriate level of cerebral perfusion in patients with sepsis is 55–115 mmHg [12]. Nonetheless, large cohort studies exploring the relationship between optimal hemodynamic parameters and SAE are lacking. Moreover, the hemodynamics of patients with SAE consuming vasopressors are unclear. Therefore, this study aims to explore the optimal hemodynamic therapy goals for macrocirculation (systolic blood pressure, diastolic blood pressure, and mean arterial pressure) and microcirculation (lactate) in patients with SAE.

## 2. Materials and Methods

### 2.1. Study Settings

We retrospectively collected data from the Medical Information Mart for Intensive Care IV (MIMIC-IV 1.0) and the multicenter database eICU Collaborative Research Database (eICU-CRD v2.0). The MIMIC-IV includes 69,619 ICU admissions from 2008 to 2019, and eICU-CRD covers 200,859 ICU admissions from 2014 to 2015 at 208 U.S. hospitals. The study team completed the Collaborative Institutional Training Initiative Examination and obtained the certification number 33690380 on 8 October 2019. The Massachusetts Institute of Technology Review Committee approved the above databases. Because this was a retrospective study, patients’ written informed consent was not required. The personal information of all patients was identified and includes demographic information, site of infection, microbiology type, vital signs, laboratory parameters, Glasgow Coma Scale (GCS), sequential organ failure assessment (SOFA) score, and other patient data. The raw data were extracted by employing structure query language (SQL) with Navicat and further processed using R software.

### 2.2. Patients

The study population was compliant with the diagnostic criteria of sepsis 3.0. [11]. We included patients with SAE that had a GCS < 15 or were diagnosed with delirium from previous literature studies based on current diagnostic criteria [3,4,5,13]. For patients undergoing sedation or surgery, the GCS scores before these procedures were extracted. Furthermore, we excluded consciousness disorders with organic causes. Additional exclusion criteria were as follows: (1) Patients with traumatic brain injury, meningitis, encephalitis intracerebral hemorrhage, cerebral embolism, ischemic stroke, epilepsy, brain tumor, intracranial infection, or any other cerebrovascular disease; (2) Patients that had mental disorders or neurological diseases; (3) Patients with a history of chronic alcohol or drug abuse; (4) Patients diagnosed with metabolic encephalopathy, hepatic encephalopathy, hypertensive encephalopathy, hypoglycemic coma, and liver or kidney diseases that affect consciousness; (5) Changes in consciousness caused by severe electrolyte imbalance or glycemic disorders, including hyponatremia (<120 mmol/L), hyperglycemia (>180 mg/dL), or hypoglycemia (<54 mg/dL); (6) Patients with missing GCS scores; (7) Patients with missing values for systolic blood pressure, diastolic blood pressure, and mean arterial pressure; (8) Patients < 18 years old.

### 2.3. Data Collection

Demographic characteristics, including age and gender, and coexisting illnesses, including hypertension, diabetes, respiratory diseases, and renal diseases, were collected for all patients. Additionally, we recorded the infection site and the microbiological infection type for sepsis patients. The worst vital signs and laboratory parameters were extracted in the hospital. Patient’s disease severity scores, including SOFA and GCS, were gathered. Moreover, data on the types of vasopressor drugs used during hospitalization and whether mechanical ventilation was used were collected. The prognostic indices of patients, including length of hospital stay, hospital mortality, and 28-day mortality were retrieved. Patients with repeated ICU admissions were excluded, and only the first admission was recorded.

### 2.4. Statistical Analysis 

The continuous variables were represented as the median and interquartile range (IQR). They showed a skewed distribution and were tested by the Shapiro–Wilk test. The Mann–Whitney U test was used to compare the continuous variables between the SAE and non-SAE groups, and the survival and non-survival groups, while the categorical variables were represented as counts and percentages and compared using Fisher’s exact test.

The LASSO regression model was used to select the characteristic variables in the high-dimensional sparse model and remove the collinearity between characteristic variables [14]. The characteristic variables related to the incidence and 28-day mortality of SAE were accurately selected by LASSO regression and multivariate analysis. A generalized additive model was used to evaluate the relationship of hemodynamic indices with the incidence and 28-day mortality in patients with SAE [15]. Finally, a multivariate logistic regression model was used to confirm the relationship between hemodynamic indices and patients with SAE. 

We used the propensity score matching to match the covariates that were different between Supplementary Materials S1 and S2, except systolic blood pressure, diastolic blood pressure, mean arterial blood pressure, lactate levels, length of hospital stay, hospital mortality, and 28-day mortality. Covariates were matched in Supplementary Materials S1 and S7. The multivariate logical expression and extreme gradient boosting (XGBoost) were used in the propensity score matching model. Furthermore, we verified the accuracy of the study results using inverse probability weighting (IPW) and doubly robust estimation [16,17]. IPW is calculated based on the propensity score matching weight. Doubly robust estimation determines whether there is a deviation in the assumptions of the multivariate logistic regression analysis and propensity score matching analysis. The doubly robust estimation model combines the multivariate logistic model and the propensity score matching model to obtain an effect estimator with double robustness. The standardized mean differences (SMD) of the original cohort were compared with those of the IPW cohorts to assess whether IPW reduces the imbalance in the distribution of the covariates. All statistical analyses were performed using R software. A *p*-value < 0.05 was considered statistically significant.

## 3. Results

### 3.1. Baseline Characteristics

**Figure 1:**
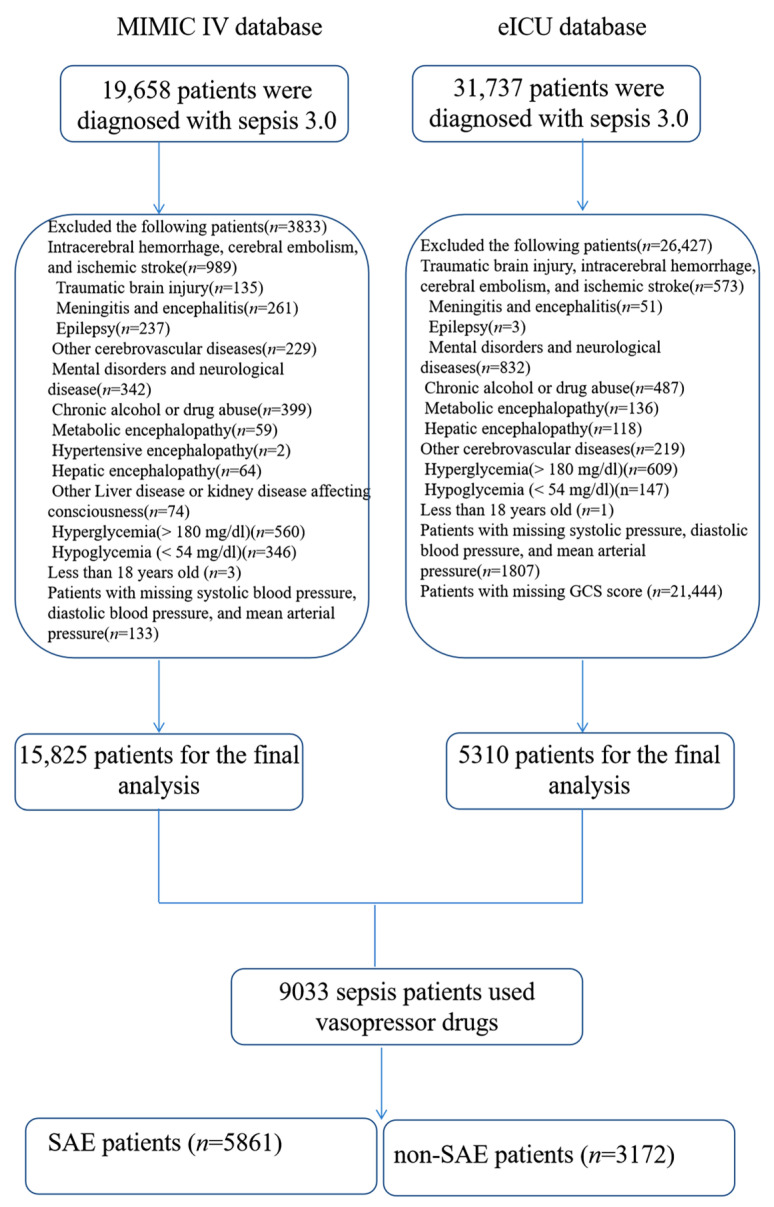
Flow chart for patient selection. SAE: sepsis-associated encephalopathy.

15,825 and 5310 patients with sepsis were extracted from the MIMIC-IV and eICU databases, respectively, based on the inclusion and exclusion criteria. There were 5861 patients (64.89%) in the SAE group and 3172 patients (35.11%) in the non-SAE group. We excluded patients with sepsis who did not use vasopressors (Figure 1).

Table 1 describes the characteristics and outcomes of the patients with sepsis in the study cohort. We observed that females and older patients were prone to SAE. More patients with SAE suffered from diabetes, renal diseases, lung infections, catheter infections, *Klebsiella* infection, *Escherichia coli* infection, *Pseudomonas aeruginosa* infection, *Acinetobacter baumannii* infection, and fungus infections than patients in the non-SAE group. Additionally, patients with SAE had higher heart and respiratory rates than those with non-SAE. Although, systolic blood pressure, diastolic blood pressure, and mean arterial pressure were lower in the SAE group than in the non-SAE group. Moreover, the patients with SAE had a higher hospital mortality, 28-day mortality, and length of hospital stay than those in the non-SAE group. In addition, more patients with SAE were treated with mechanical ventilation.

The original cohort study identified differences in many variables between the SAE and non-SAE groups. Furthermore, in order to observe the differences between the SAE and non-SAE groups in terms of systolic blood pressure, diastolic blood pressure, mean arterial pressure, and lactate levels, we matched 25 covariates with *p* < 0.05 in the original cohort, excluding systolic blood pressure, diastolic blood pressure, mean arterial pressure, lactate levels, length of hospital stay, hospital mortality, and 28-day mortality (Supplementary Materials S1). After matching, low levels of systolic blood pressure, diastolic blood pressure, and mean arterial pressure, as well as high levels of lactate, were identified as risk factors for SAE (Table 1).

### 3.2. Screening Characteristic Variables for SAE Incidence

**Figure 2:**
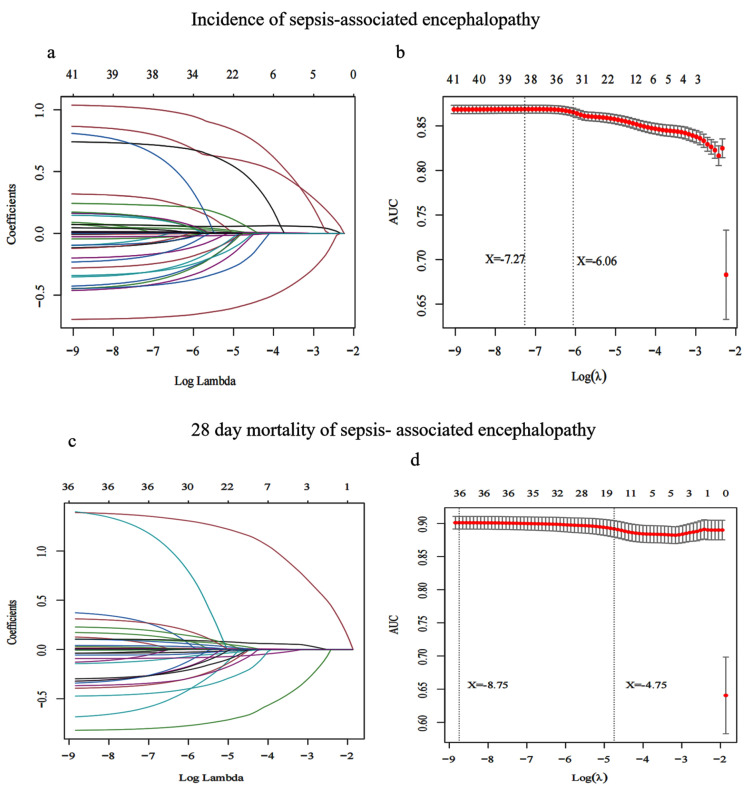
LASSO regression was used to screen the characteristic variables associated with the incidence and 28-day mortality of sepsis-associated encephalopathy. (**a**,**c**) The punishment to the model increases with an increase in the log lambda value, such that fewer variables are included in the model. (**b**,**d**) The dotted line on the left indicates the inclusion of independent variables in the minimum model, while the dotted line on the right indicates the inclusion of independent variables in the most streamlined model.

The SAE and non-SAE groups showed significant differences in many variables, and there was collinearity between many of them (Table 1). Using LASSO regression, 38 characteristic variables of the minimum model and 34 characteristic variables of the most streamlined model with a *p* < 0.05 in Table 1 were selected (Figure 2b). The minimum model log (λ) was −7.27 and the streamlined model log (λ) was −6.06. 

**Figure 3:**
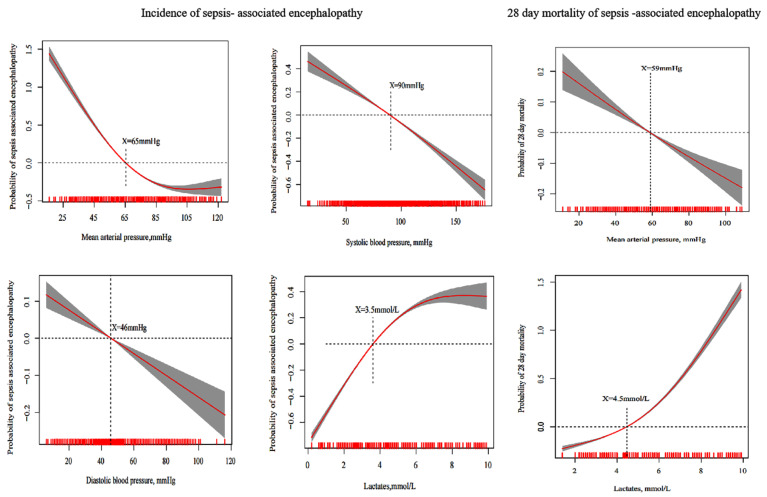
Generalized additive model evaluates the relationship between hemodynamic parameters and the incidence and 28-day mortality in sepsis-associated encephalopathy.

We selected the characteristic variables of the streamlined model for further analysis (Supplementary Materials S2). The generalized additive model demonstrated a linear relationship between hemodynamic indices and the incidence of SAE. We found that a systolic blood pressure < 90 mmHg, a diastolic blood pressure < 46 mmHg, a mean arterial blood pressure < 65 mmHg, and lactate levels > 3.5 mmol/L can increase the incidence of SAE (*p* < 0.001) (Figure 3).

### 3.3. Multiple Model Analysis of Risk Factors for SAE Incidence 

The consistent results of multivariate logistic analysis, propensity score matching, IPW, and doubly robust estimation showed that a systolic blood pressure ≥ 90 mmHg, a mean arterial blood pressure ≥ 65 mmHg, and lactate levels ≤ 3.5 mmol/L were protective factors against the incidence of SAE. After propensity matching, we found differences in systolic blood pressure, diastolic blood pressure, mean arterial blood pressure, and lactate levels between the SAE and non-SAE groups. Diastolic blood pressure was not supported by the multivariate logistic analysis, propensity matching score, IPW, and doubly robust estimation model (Table 2 and Supplementary Materials S3). The SMD of the original cohort was compared with those of the IPW cohorts, which showed that almost all variables had SMD values < 10% and the matching effect was good (Supplementary Materials S1). The covariates of the doubly robust estimation are all the variables shown in Supplementary Materials S1.

### 3.4. Screening Characteristic Variables for 28-Day Mortality of SAE

We divided the patients with SAE into survival (*n* = 5022) and non-survival groups (*n* = 839) (Supplementary Materials S4). The results of Supplementary Materials S4 showed that the survival and non-survival groups had significant differences (i.e., *p* < 0.05) in many characteristic variables. Using LASSO regression, 36 characteristic variables of the minimum model and 19 characteristic variables of the most streamlined model having *p* < 0.05 were selected (Supplementary Materials S5) (Figure 2d). The streamlined model log (λ) was −8.75, and the streamlined model log (λ) was −4.75.

### 3.5. Generalized Additive Model to Estimate the Optimal Hemodynamic Targets for 28-Day Mortality of SAE

We selected the characteristic variables of the streamlined model for further analysis. Systolic blood pressure was removed from the results of the LASSO regression analysis in the minimum model. The generalized additive model showed a linear relationship between mean arterial pressure and lactate levels with a 28-day mortality of SAE. We found that a mean arterial pressure ≥ 59 mmHg and lactate levels ≤ 4.5 mmol/L reduce the 28-day mortality in patients with SAE (Figure 3). 

### 3.6. Multiple Model Analysis of Risk Factors for SAE Hospital Mortality

The multivariate logistic analysis, propensity score matching, IPW, and doubly robust estimation showed that a mean arterial pressure ≥ 59 mmHg and lactate levels ≤ 4.5 mmol/L were independent protective factors against 28-day mortality (Table 3 and Supplementary Materials S6). The SMD of the original cohort was compared with those of the IPW cohorts, which showed that all variables had SMD values < 10% and the matching effect was good (Supplementary Materials S7). The covariates of doubly robust estimation are all the variables shown in Supplementary Materials S7.

## 4. Discussion

Cerebral perfusion is closely related to microcirculation and microcirculation. Many researchers have proposed cerebral perfusion impairment and paid tremendous attention to the pathogenesis of SAE [7,8,18]. For instance, Marion Griton et al., found significantly decreased cerebral blood perfusion in rats with septic neurological dysfunction induced by cecal ligation and puncture [19]. In addition, autopsies of patients who died of sepsis showed varying degrees of ischemic changes in multiple regions of the brain [20]. The mean arterial pressure is crucial to the mean systemic filling pressure, which drives venous return and cardiac output. Thus, increasing the mean arterial pressure increases blood flow to the tissues and tissue perfusion. The mean arterial pressure is associated with systolic blood pressure and diastolic blood pressure. Although the brain can auto-regulate blood flow, mean arterial pressure less than a certain threshold is associated with decreased organ perfusion [21]. The Surviving Sepsis Campaign (SSC) guidelines recommend targeting a mean arterial pressure of > 65 mmHg for initial resuscitation. However, it is unclear whether this blood pressure treatment is effective for patients with SAE. Therefore, this cohort study explored the appropriate mean arterial pressure treatment targets for patients with SAE.

Previous studies showed that patients with sepsis have a high incidence of encephalopathy [2,22,23]. Consistent with previous work, this study found that the incidence of SAE was 64.89%. In addition, patients with SAE had higher SOFA scores and more patients used mechanical ventilation, indicating that they had more organ dysfunction and serious disease than those with non-SAE. Moreover, we confirmed that patients with SAE had longer hospital stays, higher hospital mortalities, and 28-day mortalities than those with non-SAE. These findings showed that patients with SAE have a poor clinical prognosis, which is consistent with the results of Feng, Q. and Chen, J., et al. [24,25]. Patients with sepsis had not only a high incidence of SAE but also a poor clinical prognosis. Therefore, we need to identify potentially modifiable factors contributing to the incidence and mortality of SAE.

This cohort study was conducted using two large databases, as well as demonstrated that blood pressure and lactate levels were associated with poor neurological outcomes of sepsis and poor prognosis of patients with SAE. A mean arterial pressure ≥ 65 mmHg, systolic blood pressure ≥ 90 mmHg, and lactate levels ≤ 3.5 mmol/L reduce the incidence of SAE, whereas lactate levels ≤ 4.5 mmol/L and a mean arterial pressure ≥ 59 mmHg reduce the 28-day mortality in patients with SAE.

This study found that a mean arterial pressure ≥ 65 mmHg was associated with the lowest incidence of SAE, which indicates that a mean arterial pressure ≥ 65 mmHg ensures cerebral perfusion. This is consistent with the SSC guidelines for recommended targeting. In addition, our cohort study identified a linear relationship between the mean arterial pressure and the 28-day mortality in patients with SAE. An optimal mean arterial pressure > 59 mmHg was associated with the lowest 28-day mortality in patients with SAE, indicating that low cerebral perfusion can increase the mortality of such patients. Considering the incidence and mortality of SAE, we recommend that the mean arterial pressure of patients with SAE should be controlled at >65 mmHg. Kathryn Rosenblatt et al., suggested that the mean arterial pressure should be controlled at >55 mmHg, considering six patients with SAE. The results of our cohort study were narrower than those of Kathryn Rosenblatt et al., Furthermore, we analyzed the effect of systolic blood pressure on microcirculation in patients with SAE. We found that a systolic blood pressure < 90 mmHg increases the incidence of SAE, therefore, a systolic blood pressure > 90 mmHg is recommended. To prove the reliability of the results, the above microcirculation parameters were supported by multivariable logistic analysis, propensity score matching, propensity score IPW, and doubly robust estimation models.

Adequate cerebral blood flow is an important factor to ensure cerebral perfusion. However, some patients can develop sepsis-associated brain dysfunction even if the overall hemodynamics seem to be sufficient [9]. Cerebral microcirculation disorder plays an important role in the pathogenesis of SAE [10,26]. Lactate is an important indicator of microcirculation and can be easily obtained and monitored by clinicians. Thus, we analyzed the relationship of lactate levels with the incidence and 28-day mortality in patients with SAE. The results revealed that lactate levels ≤ 3.5 mmol/L might reduce the incidence and 28-day mortality in patients with SAE. Elizabeth M. et al., found that the timely monitoring of changes in lactate levels is closely related to the survival rate in patients with sepsis [27]. The relationship between lactate levels and the mortality in patients with sepsis has been well-established [28,29]. This study showed that lactate levels play an important role in reducing the incidence and mortality in patients with SAE. We suggest that lactate levels should be controlled at <3.5 mmol/L for patients with SAE. Nonetheless, the relationship between microcirculation and SAE requires additional microcirculation indicators, such as central venous oxygen saturation and others.

## 5. Limitations

Inevitably, the study has certain limitations: (1) as it was a retrospective observational study, the results of this work can only explain an association, rather than a causal relationship, between the hemodynamic indices and SAE; (2) although we used LASSO regression, logistic regression, propensity score matching, propensity score IPW, and doubly robust estimation to remove confounding factors, there were many other confounding factors that may cause information bias and deviation from the study results; (3) the definition of SAE in this study refers to the methods of previous high-quality retrospective studies on SAE, and the lack of a gold standard for SAE diagnosis may lead to the inaccurate selection of patients with SAE. 

Despite these limitations, the large cohort study of multiple models provides a reference value for clinicians in the hemodynamic management of patients with SAE. At last, this study used only a single value for blood pressure and lactate values in patients with sepsis, whereas it may be more meaningful to observe their dynamic changes.

## 6. Conclusions

We should implement better management of blood pressure and lactate levels in patients with SAE, especially for those that need vasopressors. We recommend that the mean arterial pressure of patients with sepsis should stay at ≥65 mmHg, lactate levels at ≤3.5 mmol/L, and systolic blood pressure at ≥90 mmHg to ensure proper cerebral perfusion and reduce the incidence and mortality in patients with SAE. It would be more meaningful, however, to dynamically monitor blood pressure and lactate levels. The results of this study provide a reference for clinicians to prevent and treat patients with SAE. Future randomized trials could also adopt the results of this study as a reference target.

## Figures and Tables

**Table 1:** Baseline characteristics and outcomes of patients with sepsis.

	Original Cohort			Match Cohort		
	Non-SAE Patients (*n* = 3172)	SAE Patients (*n* = 5861)	*p*	Non-SAE Patients (*n* = 2777)	SAE Patients (*n* = 2777)	*p*
Baseline variables						
Age (years) (median [IQR])	68.00 [60.00, 76.00]	70.00 [61.00, 79.00]	<0.001	69.00 [60.00, 77.00]	67.00 [58.00, 77.00]	<0.001
Gender, (M (%))	2115 (66.7)	3475 (59.3)	<0.001	1801 (64.9)	1824 (65.7)	0.535
Coexisting illness, (*n* (%))						
Hypertension	478 (15.1)	895 (15.3)	0.823	437 (15.7)	391 (14.1)	0.09
Diabetes	645 (20.3)	1356 (23.1)	0.002	599 (21.6)	529 (19.0)	0.021
Respiration	751 (23.7)	1383 (23.6)	0.953	724 (26.1)	391 (14.1)	<0.001
Renal	1272 (40.1)	2578 (44.0)	<0.001	1106 (39.8)	1067 (38.4)	0.296
Site of infection, (*n* (%))						
Urinary	214 (6.7)	440 (7.5)	0.197	197 (7.1)	197 (7.1)	1
Lung	128 (4.0)	391 (6.7)	<0.001	120 (4.3)	140 (5.0)	0.227
Catheter	20 (0.6)	110 (1.9)	<0.001	20 (0.7)	23 (0.8)	0.759
Skin and soft tissue	111 (3.5)	214 (3.7)	0.756	99 (3.6)	90 (3.2)	0.554
Abdominal cavity	91 (2.9)	189 (3.2)	0.385	88 (3.2)	77 (2.8)	0.429
Microbiology type, (*n* (%))						
Acinetobacter baumannii	3 (0.1)	21 (0.4)	0.035	3 (0.1)	2 (0.1)	1
Klebsiella	139 (4.4)	355 (6.1)	0.001	130 (4.7)	114 (4.1)	0.326
Escherichia Coli	263 (8.3)	683 (11.7)	<0.001	256 (9.2)	213 (7.7)	0.043
Pseudomonas aeruginosa	85 (2.7)	244 (4.2)	<0.001	82 (3.0)	59 (2.1)	0.061
Staphylococcus aureus	652 (20.6)	1283 (21.9)	0.147	575 (20.7)	510 (18.4)	0.03
Fungus	240 (7.6)	914 (15.6)	<0.001	238 (8.6)	187 (6.7)	0.012
Vital signs, (median [IQR])						
Heart rate (bpm)	94.00 [86.00, 107.00]	98.00 [88.00, 113.00]	<0.001	95.00 [87.00, 108.00]	95.00 [86.00, 108.00]	0.082
Respiratory rate (bpm)	25.25 [22.00, 29.00]	26.00 [22.50, 31.00]	<0.001	26.00 [22.00, 30.00]	25.00 [21.00, 29.00]	<0.001
Systolic blood pressure (mmHg)	90.00 [82.00, 101.00]	86.00 [78.00, 95.00]	<0.001	92.00 [85.00, 102.00]	90.00 [82.00, 101.00]	0.026
Diastolic blood pressure (mmHg)	45.00 [40.00, 52.00]	45.00 [39.00, 51.00]	<0.001	49.00 [43.00, 56.00]	45.00 [40.00, 52.00]	<0.001
Mean arterial pressure (mmHg)	66.00 [63.00, 69.00]	58.00 [53.00, 64.00]	<0.001	66.00 [63.00, 69.00]	64.00 [59.00, 71.00]	<0.001
Laboratory parameters (median [IQR])						
White blood cell (×10^9^ /L)	14.80 [10.90, 19.60]	14.60 [10.70, 19.60]	0.241	14.70 [10.90, 19.82]	14.20 [10.50, 18.80]	<0.001
Hemoglobin (g/dL)	9.30 [8.10, 10.60]	9.20 [7.90, 10.60]	0.004	9.30 [8.10, 10.60]	9.50 [8.20, 10.90]	<0.001
Platelet (×10^9^ /L)	137.00 [105.00, 189.00]	147.00 [105.00, 209.00]	<0.001	138.00 [105.00, 193.00]	149.00 [110.00, 201.00]	<0.001
INR	1.40 [1.30,1.70]	1.50 [1.20, 1.70]	0.026	1.40 [1.30, 1.70]	1.40 [1.20, 1.69]	0.119
PT(s)	15.70 [14.10, 18.30]	16.10 [14.00, 18.70]	0.070	15.80[14.10, 18.30]	15.80 [13.90, 18.20]	0.192
PTT(s)	35.20 [30.20, 42.12]	36.80 [30.50, 44.30]	<0.001	35.50[30.30, 42.90]	35.60 [30.10, 42.90]	0.635
Creatinine (mg/dL)	1.10 [0.80, 1.60]	1.10 [0.80, 1.70]	0.183	1.10 [0.80, 1.68]	1.05 [0.80, 1.50]	0.005
Blood urea nitrogen (mg/dL)	20.00 [15.00, 31.00]	22.00 [15.00, 36.00]	<0.001	20.00 [15.00, 32.00]	20.00 [15.00, 33.00]	0.655
Albumin (g/dL)	3.60 [2.90, 4.20]	3.40 [2.70, 4.00]	<0.001	3.60 [2.80, 4.20]	3.40 [2.70, 4.10]	<0.001
Glucose (mg/dL)	130.00 [111.00, 162.00]	137.00 [114.00, 174.00]	<0.001	131.00 [111.00, 163.00]	135.00 [113.00, 169.00]	0.013
Sodium (mmol/L)	139.00 [136.00, 141.00]	139.00 [137.00, 142.00]	<0.001	139.00 [136.00, 141.00]	139.00 [136.00, 141.00]	0.602
Lactates (mmol/L)	3.30 [1.90, 4.20]	4.40 [4.00, 4.80]	<0.001	3.30 [1.90, 4.20]	4.40 [3.80, 4.80]	<0.001
The score system, (median [IQR])						
SOFA	4.00 [3.00, 6.00]	6.00 [4.00, 9.00]	<0.001	5.00 [3.00, 7.00]	5.00 [3.00, 7.00]	0.001
GCS	15.00 [15.00, 15.00]	12.00 [7.00, 14.00]	<0.001	15.00 [15.00, 15.00]	13.00 [10.00, 14.00]	<0.001
Mechanical ventilation, (*n* (%))	2344 (73.9)	4617 (78.8)	<0.001	2079 (74.9)	1989 (71.6)	0.007
Use of vasopressors, (*n* (%)						
Epinephrine, (*n* (%))	478 (15.1)	888 (15.2)	0.942	456 (16.4)	274 (9.9)	<0.001
Phenylephrine, (*n* (%))	1952 (61.5)	3602 (61.5)	0.958	1685 (60.7)	1673 (60.2)	0.763
Dobutamine, (*n* (%))	122 (3.8)	204 (3.5)	0.407	101 (3.6)	80 (2.9)	0.131
Dopamine, (*n* (%))	180 (5.7)	359 (6.1)	0.414	159 (5.7)	127 (4.6)	0.06
Norepinephrine, (*n* (%))	1319 (41.6)	2954 (50.4)	<0.001	1190 (42.9)	1261 (45.4)	0.059
Length of hospital stays, days (median [IQR])	2.30 [1.30, 5.00]	4.20 [2.10, 9.70]	<0.001	2.30 [1.30, 5.10]	4.10 [1.80, 10.00]	<0.001
Hospital mortality, (*n* (%))	226 (7.1)	898 (15.3)	<0.001	213 (7.7)	267 (9.6)	0.011
28-day mortality	214 (6.7)	839 (14.3)	<0.001	0.07 (0.26)	203 (7.3)	<0.001

GCS: Glasgow coma scale; SOFA: sequential organ failure assessment; INR: international normalized ratio; PT: prothrombin time; PTT: Partial thromboplastin time.

**Table 2:** Multiple models analysis of hemodynamic index to incidence in sepsis with encephalopathy.

Models	OR	CI		*p*
		2.5%	97.5%	
**Lasso regression + Multivariate Logistic analysis (Original cohort)**				
Mean arterial pressure ≥ 65 mmHg	0.26	0.23	0.30	<0.001
Diastolic blood pressure ≥ 46 mmHg	0.94	0.83	1.05	0.258
Systolic blood pressure ≥ 90 mmHg	0.60	0.53	0.68	<0.001
Lactates ≤ 3.5 (mmol/L)	0.20	0.18	0.23	<0.001
**Propensity score matching**				
Mean arterial pressure ≥ 65 mmHg	0.34	0.30	0.38	<0.001
Diastolic blood pressure ≥ 46 mmHg	0.98	0.88	1.08	0.648
Systolic blood pressure ≥ 90 mmHg	0.60	0.54	0.66	<0.001
Lactates ≤ 3.5 (mmol/L)	0.29	0.26	0.32	<0.001
**Propensity score IPW**				
Mean arterial pressure ≥ 65 mmHg	0.33	0.30	0.36	<0.001
Diastolic blood pressure ≥ 46 mmHg	0.93	0.85	1.02	0.122
Systolic blood pressure ≥ 90 mmHg	0.64	0.58	0.70	<0.001
Lactates ≤ 3.5 (mmol/L)	0.30	0.27	0.33	<0.001
**Doubly robust** **estimation with all covariates**				
Mean arterial pressure ≥ 65 mmHg	0.63	0.60	0.67	<0.001
Diastolic blood pressure ≥ 46 mmHg	0.97	0.93	1.00	0.063
Systolic blood pressure ≥ 90 mmHg	0.86	0.83	0.89	<0.001
Lactates ≤ 3.5(mmol/L)	0.63	0.59	0.66	<0.001

**Table 3:** Multiple models analysis of hemodynamic index to 28-day mortality in sepsis with encephalopathy.

Models	OR	CI		*p*
		2.5%	97.5%	
**Lasso regression + Multivariate Logistic analysis (Original cohort)**				
Mean arterial pressure ≥ 59 mmHg	0.79	0.64	0.97	0.023
Lactates ≤ 4.5 (mmol/L)	0.068	0.055	0.084	<0.001
**Propensity score matching**				
Mean arterial pressure ≥ 59 mmHg	0.75	0.62	0.91	<0.003
Lactates ≤ 4.5 (mmol/L)	0.35	0.29	0.43	<0.001
**Propensity score IPW**				
Mean arterial pressure ≥ 59 mmHg	0.73	0.62	0.86	<0.001
Lactates ≤ 4.5 (mmol/L)	0.33	0.28	0.39	<0.001
**Doubly robust estimation** **with all covariates**				
Mean arterial pressure ≥ 59 mmHg	0.79	0.69	0.90	0.001
Lactates ≤ 4.5 (mmol/L)	0.50	0.44	0.57	<0.001

## Data Availability

The MIMIC IV database (version 1.0) is publicly available at https://mimic-iv.mit.edu/ (accessed on 21 November 2022) and the eICU database is publicly available at https://eicu-crd.mit.edu/about/eicu/ (accessed on 21 November 2022). Any studyer who adheres to the data use requirements is permitted access to these databases.The codes are available at https://github.com/MIT-LCP/mimic-iv.

## Supplementary Materials

The following supporting information can be downloaded at: https://www.mdpi.com/article/10.3390/jpm12121967/s1, Supplementary Materials S1: The standardized mean differences of the original cohort were compared with those of the IPW cohorts in sepsis patients. SMD: standardized mean differences; SOFA: sequential organ failure assessment; INR: international normalized ratio; PT: prothrombin time; PTT: partial thromboplastin time. Supplementary Materials S2: The characteristic variable of the streamline model of the incidence of sepsis-associated encephalopathy by Lasso regression modelling. SOFA: sequential organ failure assessment; INR: international normalized ratio; PT: prothrombin time; PTT: partial thromboplastin time. Supplementary Materials S3: Multivariate logistic analysis of risk factors to the incidence in patients with sepsis-associated encephalopathy. Supplementary Materials S4: Baseline characteristics and outcomes of patients with sepsis encephalopathy. Supplementary Materials S5: The characteristic variable of the streamline model of 28-day mortality of sepsis-associated encephalopathy by Lasso regression modelling. GCS: Glasgow coma scale; SOFA: sequential organ failure assessment; INR: international normalized ratio; PT: prothrombin time; PTT: partial thromboplastin in time. Supplementary Materials S6: Multivariate Logistic analysis of risk factors for 28-day mortality in patients with sepsis-associated encephalopathy. Supplementary Materials S7: The standardized mean differences of the original cohort were compared with those of the IPW cohorts in sepsis-associated encephalopathy patients. SMD: standardized mean differences; SOFA: sequential organ failure assessment; INR: international normalized ratio; PT: prothrombin time; PTT: partial thromboplastin in time.

## Abbreviations

GCS	Glasgow coma scale
MIMIC-IV	Medical Information Mart for Intensive Care IV
ICU	Intensive care unit
INR	International normalized ratio
PT	Prothrombin time
PTT	Partial thromboplastin time
SOFA	Sequential organ failure assessment
SAE	Sepsis-associated encephalopathy
IPW	Inverse probability weighting
SMD	Standardized mean differences
SSC	Surviving Sepsis Campaign
