# Supplementary material for: New Strategies to Optimize Hemodynamics for Sepsis-Associated Encephalopathy
Source: J Pers Med. 2022 Nov 28;12(12):1967. doi: 10.3390/jpm12121967 (PMC9784429; doi:10.3390/jpm12121967)
Supplement: Supplementary file 1 [file jpm-12-01967-s001.zip › Supplementary material S3.pdf]

Supplementary material S3 Multivariate logistic analysis of risk factors to the incidence in patients with sepsis associated encephalopathy

		P	OR	95.0% CI	
				Lower	Upper
Age(years)		<0.001	1.008	1.004	1.012
Gender, (n (%))					
	Female	<0.001	0.704	0.631	0.784
	Male				
Coexisting illness, (n (%))					
	Diabetes	0.077	1.118	0.988	1.266
	Renal	<0.001	0.653	0.579	0.737
Microbiology type, (n (%))					
	Escherichia Coli	0.100	1.161	0.972	1.386
	Fungus	<0.001	1.553	1.298	1.859
	Klebsiella	0.618	1.063	0.835	1.354
	Pseudomonas aeruginosa	0.235	1.196	0.890	1.608
	Acinetobacter baumannii	0.270	2.058	0.571	7.413
Vital signs					
Respiratory rate(bpm)		0.312	1.004	0.996	1.012
Heart rate (bpm)		0.002	1.004	1.002	1.007
Systolic blood pressure ≥90(mmHg)		0.146	0.920	0.822	1.030

Mean arterial pressure≥65(mmHg)	<0.001	0.244	0.215	0.276
Diastolic blood pressure≥46(mmHg)	<0.001	0.627	0.558	0.704
Laboratory parameters				
Hemoglobin(g/dL)	<0.001	1.077	1.049	1.107
INR	0.001	0.919	0.874	0.967
PTT(s)	0.302	1.001	0.999	1.003
Blood urea nitrogen (mg/dL)	0.788	1.000	0.997	1.002
Sodium (mmol/l)	<0.001	1.046	1.035	1.058
Albumin(g/dL)	<0.001	0.829	0.771	0.891
Lactates≤3.5 (mmol/L)	<0.001	0.340	0.305	0.379
Norepinephrine , (n (%))	0.009	0.851	0.755	0.960
Mechanical ventilation, (n (%))	0.001	1.249	1.100	1.418
SOFA	<0.001	1.283	1.255	1.311

SOFA: sequential organ failure assessment. INR: international normalized ratio; PT: prothrombin time.
