# Supplementary material for: New Strategies to Optimize Hemodynamics for Sepsis-Associated Encephalopathy
Source: J Pers Med. 2022 Nov 28;12(12):1967. doi: 10.3390/jpm12121967 (PMC9784429; doi:10.3390/jpm12121967)
Supplement: Supplementary file 1 [file jpm-12-01967-s001.zip › Supplementary material S4.pdf]

Supplementary material S4 Baseline characteristics and outcomes of patients with sepsis encephalopathy

	Original cohort			Match cohort		
	Survival patients (n=5022)	Non-Survival patients (n= 839)		Survival patients (n=829)	Non-Survival patients (n=829)	P
Baseline variables						
Age(years) (median [IQR])	70.00[61.00, 78.00]	73.00[61.00, 82.00]	<0.001	71.00 [62.00, 81.00]	73.00 [61.00, 82.00]	0.333
Gender, M (%)	3024 (60.2)	451 (53.8)	<0.001	441 (53.2)	448 (54.0)	0.768
Coexisting illness, (n (%))						
Hypertension	820 (16.3)	75 (8.9)	<0.001	81 (9.8)	75 (9.0)	0.674
Diabetes	1205 (24.0)	151 (18.0)	<0.001	137 (16.5)	147 (17.7)	0.557
Respiration	1219 (24.3)	164 (19.5)	0.003	177 (21.4)	161 (19.4)	0.361
Renal	2065 (41.1)	513 (61.1)	<0.001	525 (63.3)	504 (60.8)	0.311
Site of infection, (n(%))						
Urinary	366 (7.3)	74 (8.8)	0.137	78 (9.4)	73 (8.8)	0.733
Lung	315 (6.3)	76 (9.1)	0.004	94 (11.3)	76 (9.2)	0.169
Catheter	93 (1.9)	17 (2.0)	0.836	20 (2.4)	17 (2.1)	0.739
Skin and soft tissue	189 (3.8)	25 (3.0)	0.307	40 (4.8)	25 (3.0)	0.076
Abdominal cavity	155 (3.1)	34 (4.1)	0.174	36 (4.3)	33 (4.0)	0.806
Microbiology type, (n (%))						
Acinetobacter baumannii	14 (0.3)	7 (0.8)	0.029	5 (0.6)	6 (0.7)	1.000
Klebsiella	310 (6.2)	45 (5.4)	0.406	50 (6.0)	44 (5.3)	0.595
Escherichia Coli	615 (12.2)	68 (8.1)	0.001	76 (9.2)	68 (8.2)	0.542
Pseudomonas aeruginosa	217 (4.3)	27 (3.2)	0.165	42 (5.1)	27 (3.3)	0.085
Staphylococcus aureus	1162 (23.1)	121 (14.4)	<0.001	120 (14.5)	121 (14.6)	1.000
Fungus	735 (14.6)	179 (21.3)	<0.001	195 (23.5)	177 (21.4)	0.317
Vital signs, (median [IQR])						
Heart rate(bpm)	97.00[87.00, 111.00]	103.00[89.00, 120.00]	<0.001	104.00 [89.00, 122.00]	103.00[89.00, 120.00]	0.616
Respiratory rate (bpm)	26.00[22.50, 30.00]	27.00[22.00, 32.00]	0.004	26.00 [22.00, 32.00]	27.00 [22.00, 32.00]	0.639
Systolic blood pressure (mmHg)	87.00[78.00, 95.00]	83.00[75.00, 92.00]	<0.001	86.00 [77.00, 100.00]	83.00 [75.00, 92.00]	<0.001
Diastolic blood pressure (mmHg)	44.00[39.00, 50.00]	45.00[37.00, 53.00]	0.312	44.00 [38.00, 53.00]	45.00 [37.00, 53.00]	0.447

Mean arterial pressure(mmHg)	58.00[53.00, 65.00]	56.00[50.00, 62.00]	<0.001	58.00 [52.00, 67.00]	56.00[51.00, 62.00]	<0.001
Laboratory parameters (median [IQR])						
White blood cell($\times10^9$ /L)	14.50[10.78, 19.40]	14.90[10.50, 21.20]	0.116	15.00 [10.70, 20.70]	14.80 [10.50, 21.20]	0.672
Hemoglobin(g/dL)	9.20 [7.90, 10.50]	9.20 [7.90, 10.80]	0.277	8.80 [7.60, 10.30]	9.20 [7.90, 10.80]	<0.001
Platelet ($\times10^9$ /L)	146.00[106.00, 205.00]	155.00[100.00, 231.50]	0.346	145.00 [92.00, 216.00]	156.00[101.00, 232.00]	0.111
INR	1.40 [1.20, 1.70]	1.60 [1.30, 2.00]	<0.001	1.50 [1.30, 1.90]	1.60 [1.30, 2.00]	0.161
PT(s)	15.80[13.90, 18.10]	18.00[14.30, 21.80]	<0.001	17.10 [14.70, 20.40]	18.00 [14.30, 21.80]	0.138
PTT(s)	36.00[30.22, 43.00]	41.80[32.00, 52.30]	<0.001	41.80 [32.30, 55.50]	41.80 [32.00, 52.60]	0.827
Creatinine (mg/dL)	1.09 [0.80, 1.60]	1.60 [1.00, 2.50]	<0.001	1.48 [1.00, 2.50]	1.60 [1.00, 2.50]	0.362
Blood urea nitrogen (mg/dL)	21.00[15.00, 33.00]	34.00[23.00, 52.00]	<0.001	31.00 [19.00, 52.00]	34.00 [23.00, 52.00]	0.055
Albumin(g/dL)	3.50 [2.90, 4.10]	2.70 [2.20, 3.30]	<0.001	2.70 [2.20, 3.30]	2.70 [2.20, 3.30]	0.93
Glucose(mg/dL)	135.00[112.00, 169.00]	158.00[124.00, 207.00]	<0.001	149.00 [118.00, 194.00]	158.00[124.00, 207.00]	0.026
Sodium (mmol/l)	139.00[137.00, 142.00]	139.00[135.00, 143.00]	0.148	139.00 [136.00, 142.00]	139.00[135.00, 143.00]	0.75
Lactates (mmol/L)	4.40 [4.00, 4.50]	5.30 [4.50, 5.70]	<0.001	4.40 [3.80, 4.70]	5.30 [4.50, 5.70]	<0.001
The score system, (median [IQR])						
SOFA	6.00 [4.00, 8.00]	9.00 [6.00, 12.00]	<0.001	9.00 [6.00, 11.00]	9.00 [6.00, 12.00]	0.527
GCS	13.00 [8.00, 14.00]	8.00 [3.50, 12.00]	<0.001	11.00 [7.00, 14.00]	8.00 [4.00, 13.00]	<0.001
Mechanical ventilation, (n (%))	4026 (80.2)	591 (70.4)	<0.001	590 (71.2)	583 (70.3)	0.746
Length of hospital stays, days (median [IQR])	4.00 [2.00, 9.00]	6.70 [3.20, 12.15]	<0.001	7.30 [3.20, 15.30]	6.70 [3.20, 12.00]	0.001

GCS: Glasgow coma scale; SOFA: sequential organ failure assessment; INR: international normalized ratio; PT: prothrombin time; PTT: Partial thromboplastin time.
