# Supplementary material for: New Strategies to Optimize Hemodynamics for Sepsis-Associated Encephalopathy
Source: J Pers Med. 2022 Nov 28;12(12):1967. doi: 10.3390/jpm12121967 (PMC9784429; doi:10.3390/jpm12121967)
Supplement: Supplementary file 1 [file jpm-12-01967-s001.zip › Supplementary material S6.pdf]

Supplementary material S6 Multivariate logistic analysis of risk factors for 28 day mortality in patients with sepsis-associated encephalopathy

	P	OR	95.0% CI	
			Lower	Upper
Age(years)	0.006	1.012	1.006	1.018
Coexisting illness, (n (%))				
Hypertension	0.014	1.420	1.075	1.875
Diabetes	0.005	1.364	1.097	1.697
Lung	0.001	1.441	1.156	1.795
Renal	0.004	1.335	1.098	1.622
Microbiology type, (n (%))				
Staphylococcus aureus	<0.001	1.720	1.365	2.167
Vital signs				
Heart rate(bpm)	<0.001	1.010	1.006	1.014
Mean arterial pressure≥59 (mmHg)	0.018	0.705	0.592	0.84
Laboratory parameters				
Blood urea nitrogen (mg/dL)	<0.001	1.008	1.005	1.012
Albumin(g/dL)	<0.001	0.422	0.375	0.475
Lactates≤4.5(mmol/L)	<0.001	0.343	0.29	0.405
The score system				
SOFA	<0.001	1.117	1.085	1.15
GCS	<0.001	0.894	0.875	0.913

Mechanical ventilation, (n (%))	0.610	1.056	0.856	1.303
Length of hospital stays, days	<0.001	0.975	0.966	0.984

GCS: Glasgow coma scale; SOFA: sequential organ failure assessment; INR: international normalized ratio; PT: prothrombin time; PTT: partial thromboplastin in time.
