# Supplementary material for: New Strategies to Optimize Hemodynamics for Sepsis-Associated Encephalopathy
Source: J Pers Med. 2022 Nov 28;12(12):1967. doi: 10.3390/jpm12121967 (PMC9784429; doi:10.3390/jpm12121967)
Supplement: Supplementary file 1 [file jpm-12-01967-s001.zip › Supplementary materials S2.pdf]

	s1
Norepinephrine	0.952710468
Lactates	0.691095922
Dopamine	0.679268107
Acinetobacter baumannii	0.358387731
Renal disease	0.209148612
Lung infection	0.199628538
Dobutamine	0.064409844
SOFA	0.057321299
Hemoglobin	0.051318874
Phenylephrine	0.048670471
Mechanical ventilation	0.048298238
Mean arterial pressure	0.046022381
INR	0.034177852
Age	0.010766056
Blood urea nitrogen	0.008660934
Respiratory rate	0.008018268
Heart rate	0.005071254
PTT	0.003377751
Fungus	0.002923506
Catheter.related infection	0
Abdomiol.cavity infection	0
Platelets	0
White blood cell	0
Creatinine	0
Glucose	0
PT	0
Lung disease	0
Sodium	-0.000664222
Systolic blood pressure	-0.013916306
GCS	-0.021982374
Urinary.infection	-0.026738744
Diastolic blood pressure	-0.031081935
Epinephrine	-0.079802657
Hypertension	-0.116024443
Gender	-0.188054068
Klebsiella	-0.224176355
Diabetes	-0.253366147
Skin.and.soft.tissue infection	-0.272425302
Pseudomonas aeruginosa	-0.285294097
Escherichia Coli	-0.340366455

Staphylococcus aureus	-0.377740375
Albumin	-0.659349375
(Intercept)	-5.958490783
