# Supplementary material for: New Strategies to Optimize Hemodynamics for Sepsis-Associated Encephalopathy
Source: J Pers Med. 2022 Nov 28;12(12):1967. doi: 10.3390/jpm12121967 (PMC9784429; doi:10.3390/jpm12121967)
Supplement: Supplementary file 1 [file jpm-12-01967-s001.zip › Supplementary materials S5.pdf]

	s1
Lactates	1.190623138
SOFA	0.072377189
Hemoglobin	0.039687253
Diastolic blood pressure	0.016998163
Renal disease	0.012548881
Blood urea nitrogen	0.00835844
Age	0.003204019
Heart rate	0.002010749
Platelets	0.000117607
Urinary infection	0
Lung infection	0
Catheter related infection	0
Abdomiol.cavity infection	0
Gender	0
White blood cell	0
Creatinine	0
Glucose	0
Sodium	0
INR	0
PT	0
PTT	0
Respiratory rate	0
Systolic blood pressure	0
Acinetobacter baumannii	0
Klebsiella	0
Escherichia Coli	0
Fungus	0
Pseudomonas aeruginosa	0
Mechanical ventilation	-0.008906728
Length of hospital stays	-0.010907997
Skin.and.soft.tissue infection	-0.01281521
Mean arterial pressure	-0.025152016
Hypertension	-0.05658603
GCS	-0.066385138
Lung disease	-0.078261782
tangniaobing	-0.1273727
Diabetes	-0.253310449
Albumin	-0.689415967
(Intercept)	-5.142786207
