# Supplementary figures and images for: New Strategies to Optimize Hemodynamics for Sepsis-Associated Encephalopathy
Source: J Pers Med. 2022 Nov 28;12(12):1967. doi: 10.3390/jpm12121967 (PMC9784429; doi:10.3390/jpm12121967)

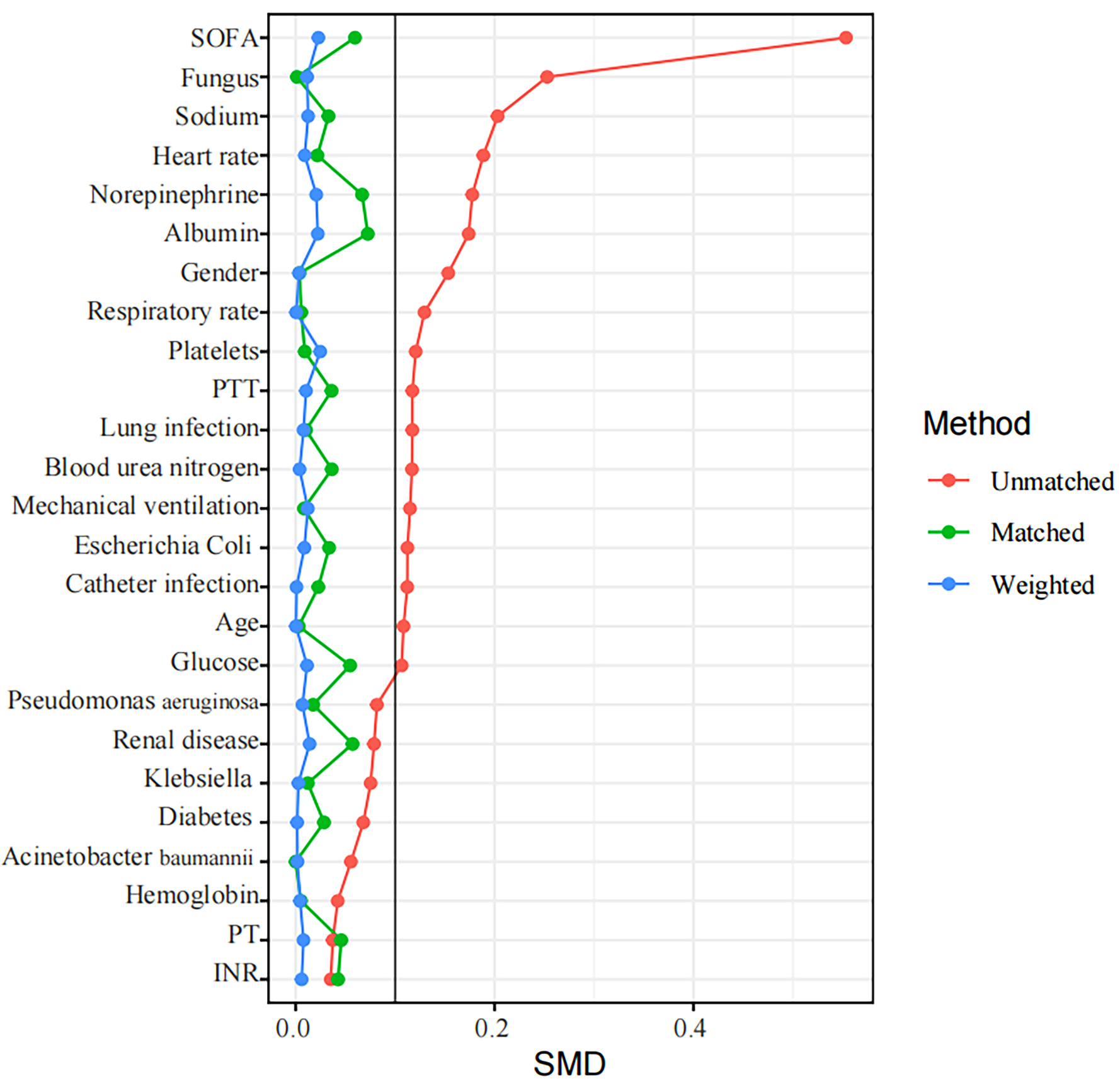

Supplement: Supplementary file 1 [file jpm-12-01967-s001.zip › Supplementary material S1.tif]

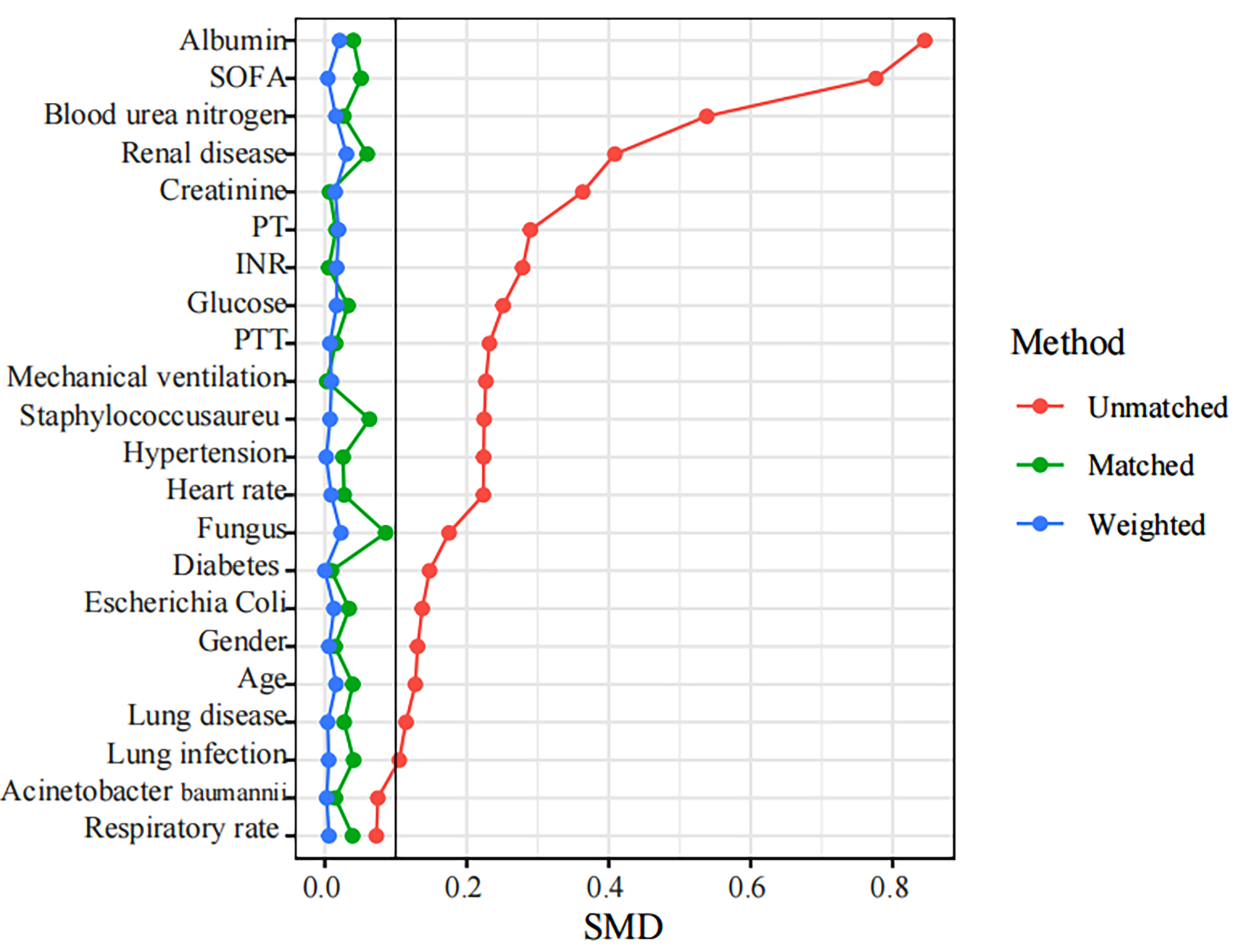

Supplement: Supplementary file 1 [file jpm-12-01967-s001.zip › Supplementary material S7.tif]
